# Diagnostic and severity scores for Cockayne syndrome

**DOI:** 10.1186/s13023-021-01686-8

**Published:** 2021-02-03

**Authors:** M. A. Spitz, F. Severac, C. Obringer, S. Baer, N. Le May, N. Calmels, V. Laugel

**Affiliations:** 1 Service de Pédiatrie Spécialisée et Générale, Unité de Neurologie Pédiatrique, Hôpital de Hautepierre, Hôpitaux Universitaires de Strasbourg, Strasbourg, France; 2 Groupe Méthode en Recherche Clinique, Service de Santé Publique, Hôpitaux Universitaires de Strasbourg, Strasbourg, France; 3 Laboratoire de Biostatistique et d’Informatique Médicale, ICube, UMR 7357, Faculté de Médecine, Hôpitaux Universitaires de Strasbourg, Strasbourg, France; 4 Laboratoire de Génétique Médicale, Institut de Génétique Médicale d’Alsace, Faculté de Médecine de Strasbourg, Hôpitaux Universitaires de Strasbourg, Strasbourg, France; 5 Laboratoire de Diagnostic Génétique, Institut de Génétique Médicale d’Alsace, Nouvel Hôpital Civil, Hôpitaux Universitaires de Strasbourg, Strasbourg, France

**Keywords:** Cockayne syndrome, Score, Diagnosis, Clinical severity

## Abstract

**Background:**

Cockayne syndrome is a progressive multisystem genetic disorder linked to defective DNA repair and transcription. This rare condition encompasses a very wide spectrum of clinical severity levels ranging from severe prenatal onset to mild adult-onset subtypes. The rarity, complexity and variability of the disease make early diagnosis and severity assessment difficult. Based on similar approaches in other neurodegenerative disorders, we propose to validate diagnostic and severity scores for Cockayne syndrome.

**Methods:**

Clinical, imaging and genetic data were retrospectively collected from 69 molecularly confirmed CS patients. A clinical diagnostic score and a clinical-radiological diagnostic score for CS were built using a multivariable logistic regression model with a stepwise variable selection procedure. A severity score for CS was designed on five items (head circumference, growth failure, neurosensorial signs, motor autonomy, communication skills) and validated by comparison with classical predefined severity subtypes of CS.

**Results:**

Short stature, enophtalmos, hearing loss, cataracts, cutaneous photosensitivity, frequent dental caries, enamel hypoplasia, morphological abnormalities of the teeth, areflexia and spasticity were included in the clinical diagnostic score as being the most statistically relevant criteria. Appropriate weights and thresholds were assigned to obtain optimal sensitivity and specificity (95.7% and 86.4% respectively). The severity score was shown to be able to quantitatively differentiate classical predefined subtypes of CS and confirmed the continuous distribution of the clinical presentations in CS. Longitudinal follow-up of the severity score was able to reflect the natural course of the disease.

**Conclusion:**

The diagnostic and severity scores for CS will facilitate early diagnosis and longitudinal evaluation of future therapeutic interventions. Prospective studies will be needed to confirm these findings.

## Introduction

Cockayne syndrome (CS) is an autosomal recessive multisystem disorder characterized by mental retardation, microcephaly, severe growth failure, sensorial impairment, cutaneous photosensitivity, dental anomalies, recognizable facial appearance with enophtalmos [1]. The incidence of CS has been estimated at 1/360,000 births in western Europe [2]. CS is a degenerative disorder resulting in progressive neurosensorial deterioration and most patients show progressive neurological dysfunction with a combination of pyramidal, extra-pyramidal, cerebellar and peripheral signs. CS is related to defective DNA repair and transcription processes and belongs to the family of Nucleotide Excision Repair (NER) disorders together with xeroderma pigmentosum (XP) and trichothiodystrophy (TTD) [3]. Recovery of RNA synthesis (RRS, decreased in CS) and unscheduled DNA synthesis (UDS, normal in CS) are the classical gold standard functional assays which are used to ascertain the diagnosis in cultured fibroblasts [4]. The two major genes responsible for the disease are *CSA*/*ERCC8* and *CSB*/*ERCC6* [5, 6]. The CS diagnosis is classically suggested by clinical criteria [7], which have been defined before the molecular era. All CS patients show similar features but a very wide variation in severity and age of onset. Most symptoms appear and worsen with time, so that CS has traditionally been classified in three severity groups. However, it is increasingly evident that CS has a continuous spectrum of severity and that there is no clear threshold between the subgroups. More recent papers proposed revised diagnostic criteria [8, 9] and new severity classification [10] to improve the recognition of the disease and prognosis accuracy, but are based on a qualitative approach. Existing diagnosis criteria for CS have shown their limits to properly detect CS patients especially at an early stage of the disease or in mild phenotypes. Moreover, there is presently no reliable quantitative clinical tool to assess the overall severity of the disease (amid a continuous spectrum of severity) and its natural course over time.

This study aims to provide a new CS diagnostic scoring system to help clinicians in early CS diagnosis which is crucial for appropriate management of the patients and genetic counseling for families. The second aim is to establish a quantitative clinical severity score for CS as this is a major issue for the development of longitudinal follow-up studies and potential therapeutic trials.

## Materials and methods

### Patients

**Fig. 1:**
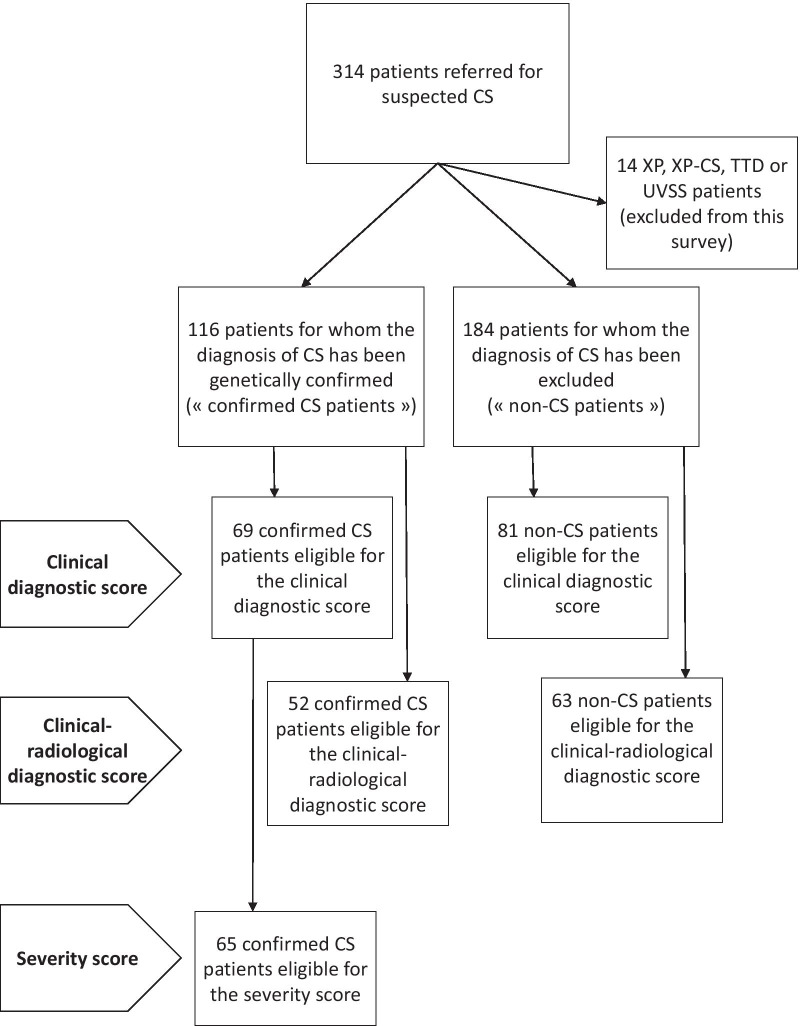
Flowchart of the patient cohort

Subjects were retrospectively selected from the database of our reference laboratory for CS which included 314 files of patients who had been referred for suspected CS and for whom the diagnosis of NER disorder had either been confirmed or disproved (Fig. 1). XP, combined XP-CS, cerebro-oculo-facio-skeletal syndrome (COFS), UV-sensitive syndrome and TTD patients were completely excluded from the analysis (n = 14). The diagnosis of CS had been confirmed in 116 patients by functional and genetic testing (“confirmed CS cases”). The absence of pathogenic variants in the NER genes and absence of functional anomaly of the NER pathway had been proved in 184 patients (“non-CS cases” serving as control cases). For the vast majority of these cases for which the diagnosis of CS had been excluded, no formal diagnosis had been reached at the time of the study. Extensive clinical evaluation at the time of the diagnosis was available for 69 patients among the genetically confirmed CS cases and for 81 patients among the non-CS cases. Extensive data for both clinical and radiological items at the time of the diagnosis were available for 52 CS cases and 63 non-CS cases. All eligible patients were included in the analysis and these groups were used to build the diagnostic scores presented thereafter. We intentionally chose to use these defined groups of non-CS patients, who resemble the CS phenotype, as control groups (and not healthy controls) to obtain more stringent criteria and sharpen our diagnostic scores. Among the group of 69 genetically confirmed CS cases, 65 patients for whom accurate and reliable assessment of the severity of growth retardation and developmental delay was available, were further selected to build the severity score. Our database and fibroblast library (DC-2014-2222) have been registered at the appropriate authority (Commission Nationale de l’Informatique et des Libertés), in accordance with relevant French laws. Full consent for genetic screening has been obtained from all families.

### Collected data

The following clinical data were collected: term and birth measurements (length, weight and head circumference), postnatal growth parameters (height, weight, head circumference), major developmental milestones, neurological symptoms at the time of the diagnosis and at different time points when available (spasticity, extra-pyramidal signs, ataxia and areflexia), dental abnormalities (dental decay, enamel hypoplasia, number and shape defects), hearing loss characteristics, ophthalmological symptoms (cataracts, retinopathy, enophthalmos), cutaneous symptoms (photosensitivity, pigmentary anomalies), age at death or last assessment. The following relevant imaging data were collected: white matter abnormalities, cerebral atrophy or enlarged lateral ventricles, cerebellar atrophy on MRI; cerebral calcifications on CT-scan (as described in Koob et al. [11]).

### Patient classification

Patients were classified according to current classifications in the literature [8–10] as follows. Type II or severe CS (CS II) is defined by the non-acquisition of independent walking, extremely limited verbal communication (few words) and early onset of the disease before the age of 3 months. Type I or moderate CS (CS I) is defined by the acquisition of independent sitting, delayed independent walking, minimal verbal communication (short sentences), good peer interactions. Type III or mild CS (CS III) is defined by the acquisition of independent walking and running (developed language, often acquired reading and writing). UVSS syndrome shares the same molecular and cellular defects as classical CS but is characterized by cutaneous photosensitivity only, without any other clinical symptom [12]. XP-CS combined phenotype is linked to mutations in XP genes and designates the combination of CS and XP signs, with functional tests showing decreased DNA repair in both RRS and UDS assays [13]. The COFS syndrome is a prenatal subgroup of CS and the most severe clinical subtype of the spectrum: it is characterized by prenatal symptoms including arthrogryposis, severe microcephaly, severe fetal growth restriction [14]. These definitions are widely and routinely used in clinical practice but it is well acknowledged that all these subgroups show obvious overlaps and that no undisputable threshold can be drawn. Among these subtypes, the rare COFS (n = 5), UVSS (n = 1) and XP-CS (n = 3) patients were excluded from the initial cohort and from our study, as defined by our inclusion criteria, since they show distinct clinical and/or genetic characteristics.

### Diagnostic scores

The present clinical diagnosis score was inspired from a previously published diagnostic scoring system developed for Niemann-Pick syndrome, which shares a similar neurodegenerative pattern with CS [15, 16]. We evaluated the presence or absence of the selected clinical symptoms detailed above in all included patients at the time of the diagnosis and we correlated these criteria to the confirmed diagnosis of CS in our reference groups of CS and non-CS patients.

In a second statistical calculation, we mixed clinical and radiological items to design a second scoring system based on both clinical and imaging criteria (designated thereafter as the clinical-radiological score).

### Severity score

The design of the severity score was based on previously published severity scoring systems used for infantile ceroid lipofuscinosis disease, which is another neurodegenerative disorder [17–19]. Based on the items already validated in this scoring system for ceroid lipofuscinosis on the one hand and on existing literature for CS on the second hand, we selected the most a priori relevant and easily available prognostic criteria and a severity score was built combining the most significant factors. The proposed score was based on 5 items including degree of microcephaly, severity of growth failure, neurosensorial examination, motor autonomy, communication skills. Each item was rated from 0 (milestone non acquired or the worst degree of severity) to 3 (normal for age) (Table 1). The validity of this severity score was then tested in our cohort by comparison with the previously defined severity groups according to classical definitions (as stated above). The severity score was calculated at least at the time of the diagnosis for each patient when possible. Longitudinal data were considered significant enough for a repeated assessment of the severity score, before and after the time of the diagnosis, when at least 5 different time points were available over at least 5 years of follow-up for type I and type III CS, and at least 3 years of follow-up for type II CS.

**Table 1:** Severity score including 5 items, each being rated from 0 (worst score) to 3 (best score)

	Score
**Head circumference**	
Normal head circumference for age	3
Head circumference between − 2 SD and − 3 SD (limits included)	2
Head circumference between − 3 SD and − 5 SD (limits excluded)	1
Head circumference equal to or below − 5 SD	0
**Weight/height**	
Absence of growth delay	3
Weight and/or height between − 2 SD and − 3 SD (limits included)	2
Weight and/or height between − 3 SD and − 5 SD (limits excluded)	1
Weight and/or height equal to or below − 5 SD	0
**Neurosensorial symptoms**	
No neurosensorial symptom	3
Neurosensorial symptoms from one or two categories^a^	2
Neurosensorial symptoms from three categories^a^	1
Neurosensorial symptoms from four categories^a^	0
**Autonomy/motor development**	
Normal motor skills for age	3
Moderate motor impairment/motor delay	2
Severe motor impairment/motor delay (or no standing position after 2 years of age)	1
No motor development/bedridden	0
**Communication**	
Normal communication and language for age	3
Moderate communication impairment/speech delay	2
Severe communication impairment (or non-verbal communication only after 2 years)	1
No communication	0
**Total**	15

*SD* standard deviation

^a^Neurosensorial categories: 1—cerebellar signs (ataxia, action tremor, cerebellar dysarthria); 2—pyramidal or extrapyramidal signs (spasticity, rigidity, akinesia); 3—peripheral nerve involvement (hyporeflexia or areflexia); 4—sensorial impairment (neurosensorial hearing loss, retinopathy, cataracts)

### Statistical analysis

A descriptive analysis was performed to present the characteristics of the cases and the controls. Categorical variables were presented as numbers and percentages. A multivariable logistic regression model was performed to create the scoring systems. The variables used in the model were all collected symptoms of CS as detailed above. A stepwise variable selection procedure based on the AIC (Akaike Information Criterion) was performed to ascertain variables associated with the disease. The regression coefficients from each variable were rounded to the nearest unit to determine a number of points assigned to the patient if the symptom is present. Finally, a predictive diagnostic score of CS based on the regression coefficients was calculated for each patient of the study. A receiver operating characteristic (ROC) analysis was carried out to investigate the performance of the score by computing the area under the curve (AUC) and its 95% confidence interval (CI) using a bootstrap resampling method (with 2000 replicates). Sensitivities and specificities of the different values of the predictive diagnostic score were computed and graphically represented to allow usable threshold selection for the classification of the risk in “low”, “moderate” or “high”. Nance and Berry criteria were also evaluated for the diagnosis of CS by computing different indicators (sensitivity, specificity, positive predictive value, negative predictive value, and exactness) with their 95% CI.

The severity score was presented using the median with the interquartile range. The normality of the distribution was assessed graphically and using Shapiro–Wilk test. Comparisons of the severity scores depending on the clinical subtype and on the involved gene were performed using beta regression models. Adjusted p-values for multiple comparisons were calculated with the “Holm” method for each model. Correlation between the age at first symptom and the severity score was estimated using Spearman coefficient correlation (ρ). A p-value < 0.05 was considered as statistically significant. Statistical analysis was performed with R software version 3.2.2 [20].

## Results

### Diagnostic scores

**Fig. 2:**
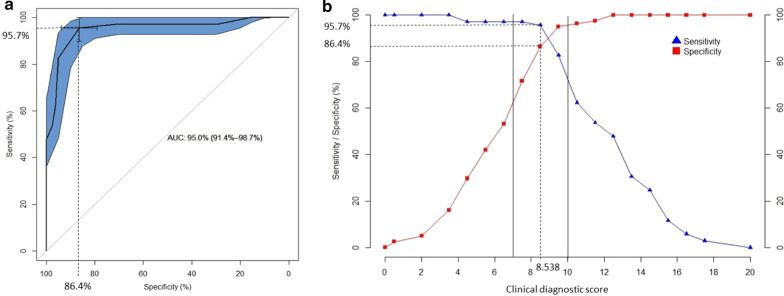
Optimal ROC curve for the clinical diagnostic score showing the maximal area under the curve (AUC) obtained for the score defined in Table 2. Optimal sensitivity and specificity on this curve are 95.7% and 86.4% respectively (**a**), corresponding to a threshold of 8.5 for the diagnostic score (**b**). Solid lines draw the thresholds of 7 and 10 that were chosen to distinguish low/moderate/high likelihood for CS diagnosis based on positive predictive value (not shown)

The multivariable logistic regression analysis applied to the groups of 69 confirmed CS cases and 81 non-CS controls led to the creation of a clinical diagnostic score based on the 10 most statistically discriminant criteria and comprised between 0 (absence of any symptom) and 20 (presence of all statistically relevant symptoms) (Table 2). From this statistical analysis, the 10 symptoms that were the most relevant and specific to distinguish genetically confirmed CS cases from non-CS cases were the following: short stature, enophtalmos, hearing loss, cataracts, cutaneous photosensitivity, frequent dental caries, enamel hypoplasia, morphological abnormalities of the teeth, areflexia and spasticity. On the ROC curve obtained for this clinical score as defined in Table 2, the optimal sensitivity and specificity of this score were 95.7% and 86.4% respectively in our cohort (Fig. 2a), which corresponds to a threshold of 8.5 for this score (Fig. 2b). The median score was 6 [4;8] for non-CS patients and was 12 [10;14] for CS patients. According to these curves, the thresholds predicting low/moderate/high likelihood for CS diagnosis were 7 and 10.

**Table 2:**
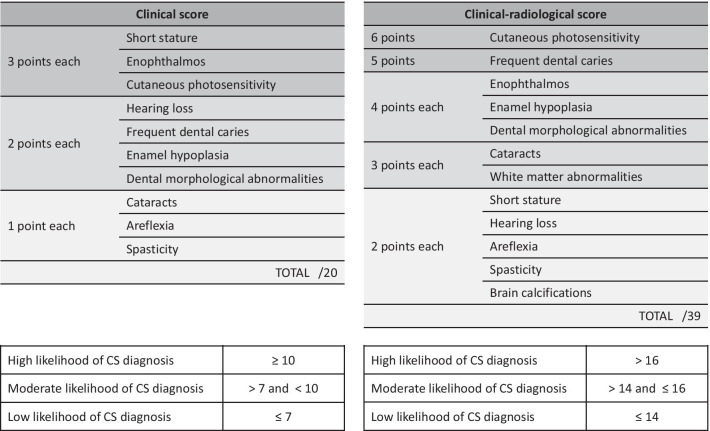
Diagnostic scores

A number of points is attributed to each symptom based on its statistical weight. The clinical score is based on 10 clinical signs. The clinical-radiological score is based on 10 clinical and 2 radiological signs. The total score is the sum of the points assigned to the symptoms observed in a given patient. High, moderate and low probability thresholds for CS diagnosis have been calculated in the defined cohort and showed below each scoring table

**Fig. 3:**
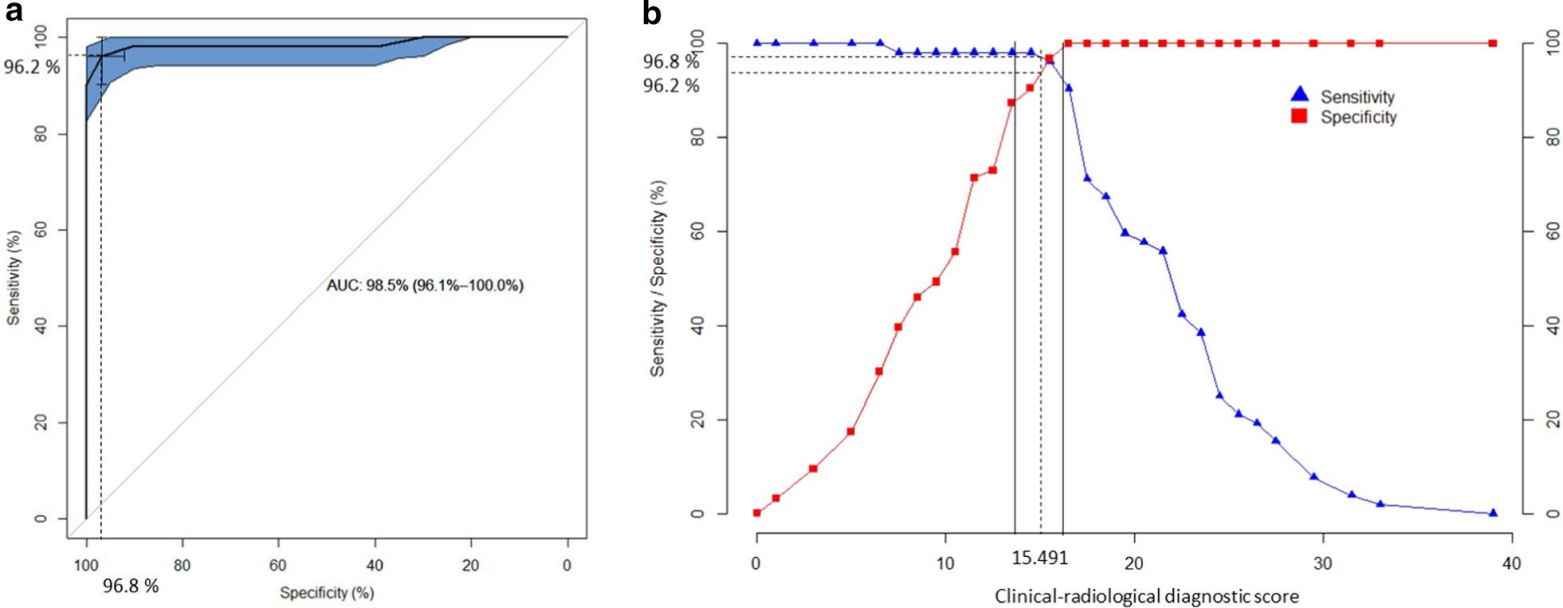
Optimal ROC curve for the clinical-radiological diagnostic score showing the maximal area under the curve (AUC) obtained for the score defined in Table 2 Optimal sensitivity and specificity on this curve are 96.2% and 96.8% respectively (**a**), corresponding to a threshold of 15.5 for the clinical-radiological diagnostic score (**b**). Solid lines draw the thresholds of 14 and 16 that were chosen to distinguish low/moderate/high likelihood for CS diagnosis based on positive predictive value (not shown)

When considering both clinical and imaging items in the groups of 52 confirmed CS cases and 63 non-CS cases, the statistical analysis led to the creation of a clinical-radiological diagnostic score calculated on 12 criteria, including the 10 clinical criteria mentioned above associated with leukodystrophy and brain calcifications which were the most statistically discriminant imaging criteria (Table 2). In our cohort, the optimal sensitivity and specificity of this clinical-radiological composite score were 96.2% and 96.8% respectively to a threshold of 15.5 (Fig. 3). The median score was 10 [6;13] for non-CS patients and 22 [17;24.25] for CS patients. The thresholds predicting low/moderate/high likelihood for CS diagnosis were 14 and 16.

In the same cohort we were also able to retrospectively test the classical Nance and Berry criteria [7] at the time of the diagnosis: the sensitivity and specificity of these criteria were only 78.8% and 88.7%. The positive predictive value of these criteria was 85.9% and the negative predictive value was 82.7%. The proposed diagnostic scoring system has thus a higher specificity and sensitivity than existing criteria.

### Severity score

**Fig. 4:**
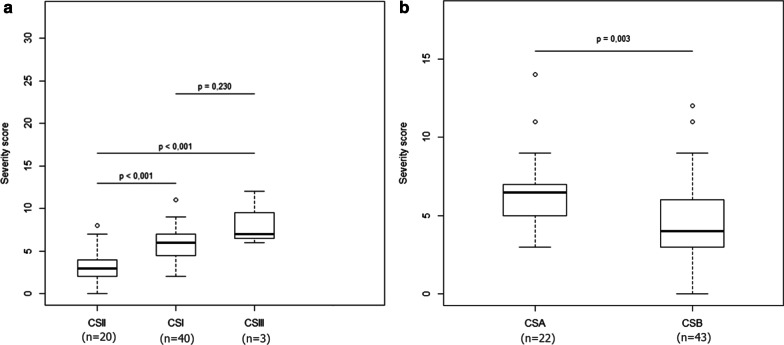
Box plots expressing severity score at the time of the diagnosis as a function of clinical subgroups (**a**) or mutated genes (**b**). Type II CS patients have a significantly lower score (more severe) at the time of the diagnosis than type I CS patients and type III CS patients. Due to the small number of type III patients available the difference between type I and type III does not reach statistical significance. CSA patients have a significantly higher (less severe) score than CSB patients at the time of the diagnosis

The 65 patient-cohort that was eligible for the clinical severity score included 40 type I CS patients, 20 type II CS patients, 3 type III CS patients and 2 CS patients of undefined subtype. The median severity score of each predefined CS subgroup was then calculated for all patients at the time of the diagnosis to validate the quantitative severity assessment provided by this score. The median score was 6 [4.75;7] for type I CS patients, 3 [2;4] for type II CS patients, 7 [6.5;9.5] for type III CS patients. The score calculated at the time of the diagnosis was shown to be able to statistically and quantitatively differentiate predefined type II and type I CS patients as well as type III and type II CS patients. Type III CS patients also scored higher than type I CS, as predicted, but due to the small number of type III patients, this difference did not reach statistical significance (Fig. 4a).

**Fig. 5:**
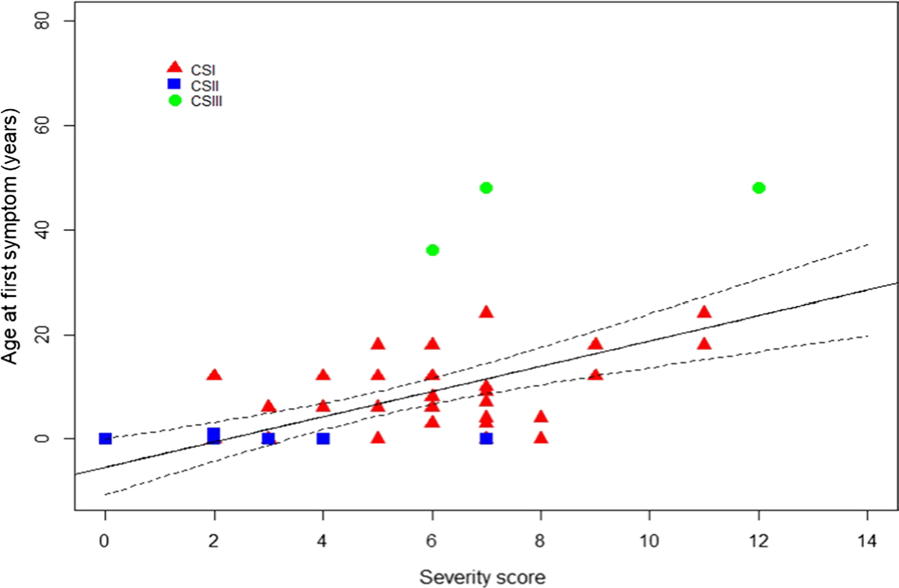
Correlation between age at first symptom and severity score at the time of the diagnosis for type I (red triangles), type II (blue squares) and type III (green circles) CS patients

Twenty-two patients presented with *CSA* mutations and 43 with *CSB* mutations. The median score was 6 [5;7] for CSA patients and was 4 [3;6] for CSB patients. The score confirmed a statistically significant difference between CSA and CSB patients in our cohort at the age of diagnosis (Fig. 4b) as suggested before in non-quantitative assessments [8]. Our results have also shown a positive correlation (rho = 0.41) between the age of the first symptom and the severity score (p < 0.01) (Fig. 5).

**Fig. 6:**
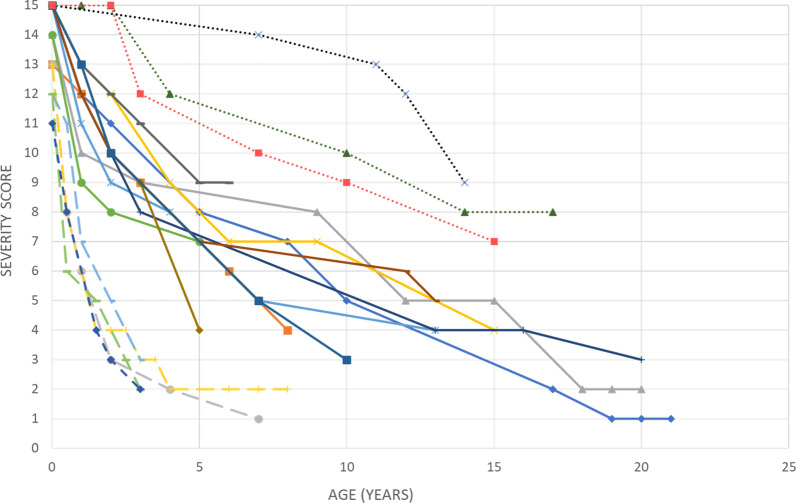
Natural course of the severity score over time in 19 CS patients. Each curve represents a different patient. Dotted curves correspond to type III CS patients, solid curves correspond to type I CS patients, dashed curves correspond to type II CS patients

Detailed longitudinal data were retrospectively available for 19 CS patients (11 predefined type I, 5 predefined type II and 3 predefined type III CS patients). Figure 6 shows the evolution of the severity score over time for these 19 individual patients. Type I CS patients show a normal score at birth and a regular decrease over time from the first year of life. Type II CS show already a slightly decreased score at birth and a rapid and dramatic decrease postnatally to reach a low plateau usually until death. Type III CS are the only patients in this series who show a normal plateau in the first years of life before a slow or delayed decline as expected.

## Discussion

The purpose of this study is to design simple, robust and quantitative scoring systems to facilitate the diagnosis and follow-up of CS patients. We have shown that the proposed scores are reliable tools in our cohort to distinguish CS from non-CS patients and to classify the levels of severity in CS in a more continuous and quantitative way than the classical overlapping subgroups. It also opens the possibility that this severity score may be used longitudinally to monitor the natural evolution of the disease and possibly the effect of future therapeutic interventions. Prospective and longitudinal studies will be necessary to validate the use of the severity score in clinical follow-up and therapeutic trials.

We acknowledge that the accuracy of both scores may have been altered by the retrospective selection of the patients, which might have biased the recruitment of the patients and the collection of the selected items. We believe however that the number and variety of patients were large enough to ensure the representativity of the cohort. The control group of non-CS patients was also not specifically designed to include all possible disorders that may be overlapping with CS but reflected the actual patients who were referred to our center and whose clinical picture was congruent with CS diagnosis according to clinical experts. We will subsequently propose that both scores be further validated through a larger use in a collaborative prospective project including other reference centers for CS worldwide.

It must be stressed that the diagnostic score detailed herein does not aim at replacing clinical expertise. The molecular analysis of the CS genes will of course remain necessary to confirm the diagnosis and the functional cellular assays of DNA repair will remain a useful and relevant complementary tool. The diagnostic score is intended to be a useful tool to help improve the diagnosis of CS and to guide the selection of adequate genetic investigations. We choose on purpose to build the diagnostic score by comparing the CS group to a control group of distinct but clinically similar non-CS disorders, and not to a normal group of healthy controls, in order to make our score more stringent for clinical use. Indeed, CS symptoms are usually easily recognized as abnormal but the delay in CS diagnosis comes from the fact that each of them may lead to many different diagnoses. This also explains that microcephaly, which is obviously a prominent and constant symptom of CS, was not considered selective enough in our statistical analysis as it is widely present in many other neurodegenerative disorders. Interestingly, microcephaly was already not considered as a major item in the Nance and Berry criteria, probably for the same reason. Our purely statistical strategy probably also led us to exclude criteria which might have been too rare in the spectrum of CS or too difficult to find by routine investigations in our cohort. Typically, pigmentary retinopathy is a quite specific feature of CS but is difficult to diagnose in young children and may have been overlooked in some patients of our cohort. Again, this diagnostic score is meant to be an easily available and useful tool for clinicians and does not intend to reflect the wholeness of the clinical picture of CS. The clinical-radiological score offers an even better specificity and sensitivity than the clinical score only but requires both a CT-scan and an MRI. In this clinical-radiological score, the respective weights of the clinical items are slightly modified as a result of the presence of the additional imaging items and possibly because a few patients used for the clinical score had not enough imaging data to be used in the clinical-radiological score. The clinical score is probably more useful for clinicians in an initial screening approach, the clinical-radiological score may be further used to investigate complex cases.

It must also be kept in mind that CS is a progressive disorder and that its cardinal symptoms develop with time. We believe that a quantitative diagnostic score, with different diagnostic likelihood thresholds, is well suited to this situation. The same diagnostic score can indeed be used at different stages during the onset of the disease and one would expect that CS patients have a higher diagnostic score after a few years of evolution: a high level of likelihood should probably be requested to consider the diagnosis of CS in patients with a longer evolution of the symptoms whereas an intermediate threshold might already be meaningful for patients who have only recently shown the first symptoms. The use of this diagnostic score in early diagnosis and the adequate likelihood threshold in this case will have to be specified by subsequent studies.

This diagnostic score is however unable to monitor the evolution of the disease after the onset of the full clinical picture and is also not intended to reflect the severity of each symptom. The severity score offers then a complementary approach to describe the severity of the disease at any given timepoint and its course over time whether it be natural or possibly modified by therapeutic interventions. The assessment of disease severity that has been captured by this severity score in our cohort at the time of diagnosis is validated by the cross analysis with the predefined clinical subgroups. This score confirms the overlap between clinical presentations and the absence of clear-cut thresholds between subgroups, even if the three classical subgroups exemplify three major profiles of natural evolution and severity among the CS spectrum. The quantitative nature of this score seems well adapted to the continuous spectrum of severity that has been suggested in previous studies on CS. Repeated assessments of the severity score is probably able to reliably and quantitatively reflect the natural course of the disease. A precise evaluation of the severity level of the disease by this score at a given time point and by its rate of deterioration could help clinicians to finely adjust the medical follow-up of the patients. The evolution of this score might also be crucial to prove the global efficiency of any therapeutic intervention in CS and would probably be a more relevant endpoint than any assessment of one single dimension of this multisystem syndrome. This scoring approach has been successfully used in ceroid-lipofuscinosis [21, 22] and very similar approaches based on functional scores are now routinely used in gene therapies for spinal muscular atrophy or Duchenne muscular dystrophy [23–26]. Recent experience in these disorders has shown that an accurate knowledge of the slope of the curves, of the interindividual variability and of the periods of plateauing is a prerequisite before using these scores in clinical trials. Prospective longitudinal studies on a larger scale will be needed to validate the use of the severity score for follow-up studies in CS and to test the potential prognostic value of the severity score.

## Data Availability

The datasets used and analysed during the current study are available from the corresponding author on reasonable request.
